# Mo Passer (Mufasa): A Cost-Effective Alternative Suture-Passer Technique for Patella Fracture Fixation

**DOI:** 10.7759/cureus.76471

**Published:** 2024-12-27

**Authors:** Nizar Ismail, Mohammed Yassin, Carl Cowin

**Affiliations:** 1 Trauma and Orthopaedics, East Midlands Deanery, Leicester, GBR; 2 Trauma and Orthopaedics, Tullamore Hospital, Tullamore, IRL; 3 Trauma and Orthopaedics, Royal Cornwall Hospital, Truro, GBR

**Keywords:** cannulated screw, fiber tape, patella fracture, patella fracture system, suture passer

## Abstract

Suture passers are indispensable instruments in orthopaedic surgery, particularly in open procedures. Commercial suture passers, while effective, can be costly and may not be readily available in all surgical settings. We present the Mo Passer (Mufasa), an innovative, cost-effective technique utilizing standard theatre materials. This paper demonstrates its application in patella fracture fixation procedures, highlighting its efficiency and economic advantages in orthopaedic surgery.

## Introduction

Patellar fractures represent 1% of all orthopaedic trauma presentations [1]. According to current epidemiological data, these fractures occur more commonly in young males (10 to 19). At older ages, they are more common among females between 60 and 80. The incidence of patellar fractures is approximately 13.1/100,000 annually [2]. The goals of surgical treatment include anatomic reduction of the articular surface, stable fixation for early mobilization, and restoration of the extensor mechanism [3].

The tension band wire technique involves fixing the fracture with two K-wires across the patella bone and supporting the fixation with the cerclage wire. In contrast, the tightrope technique is a suture technique used to support the fracture after initial fixation with cannulated screws. The suture usually passes through the screws to reinforce the fixation and add more compression. Tension band wiring and tightrope fixation had similar results regarding fracture gapping and failure. However, tightrope fixation has a lower risk of complications in managing transverse patella fractures, such as implant migration and soft tissue irritation [4,5].

Arhtrex describes a patella fracture system that provides a different approach to fixing the patella fracture from traditional K-wires in which initial fixation is achieved with two cannulated screws, and the fixation is augmented with a FiberTape suture that passes through the screws. This system requires a special suture passer that is only available in the Arthrex patella fracture system set, which limits the usage to only clinical settings with access to the Arthrex system [6].

Currently, many of the contemporary fixation techniques utilize a different set of approaches. Studies on the cannulated screws with tension band augmentation implants have had good results, reporting higher implant stability and less need for hardware removal than traditional techniques. However, such advanced techniques often necessitate special instruments for the suture placement and administration, which increases the cost of the procedure. This method is most suitable for simple transverse fractures; patella fractures with extensive comminution may be more appropriate to treat using other methods, such as plate fixation [5,7].

## Technical report

**Figure 1:**
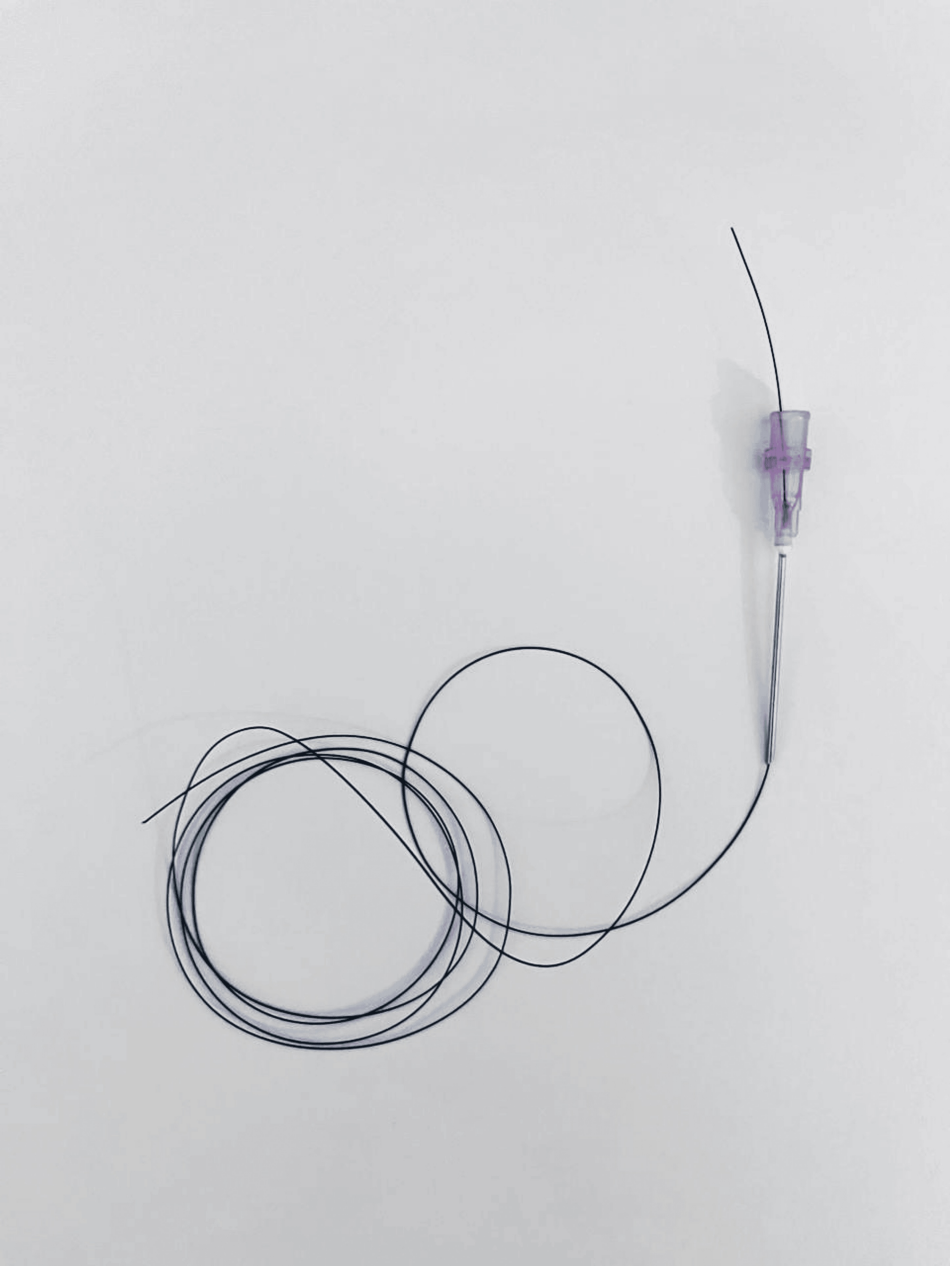
The first step of forming the suture passer: passing on the end of the 2/0 nylon stitch through an 18-gauge needle.

**Figure 2:**
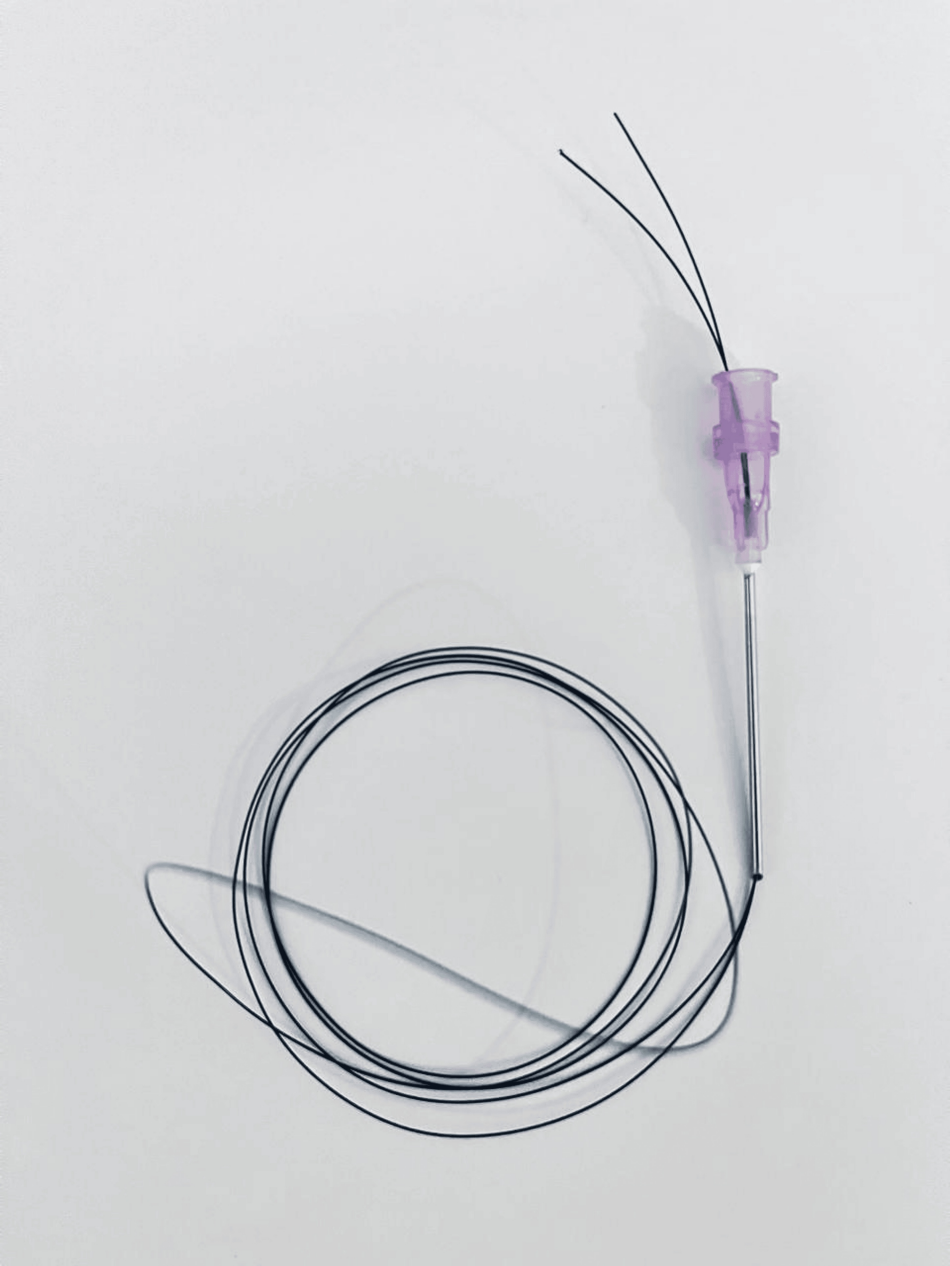
The second step of forming the suture passer: passing on the other end of the 2/0 nylon stitch through an 18-gauge needle.

**Figure 3:**
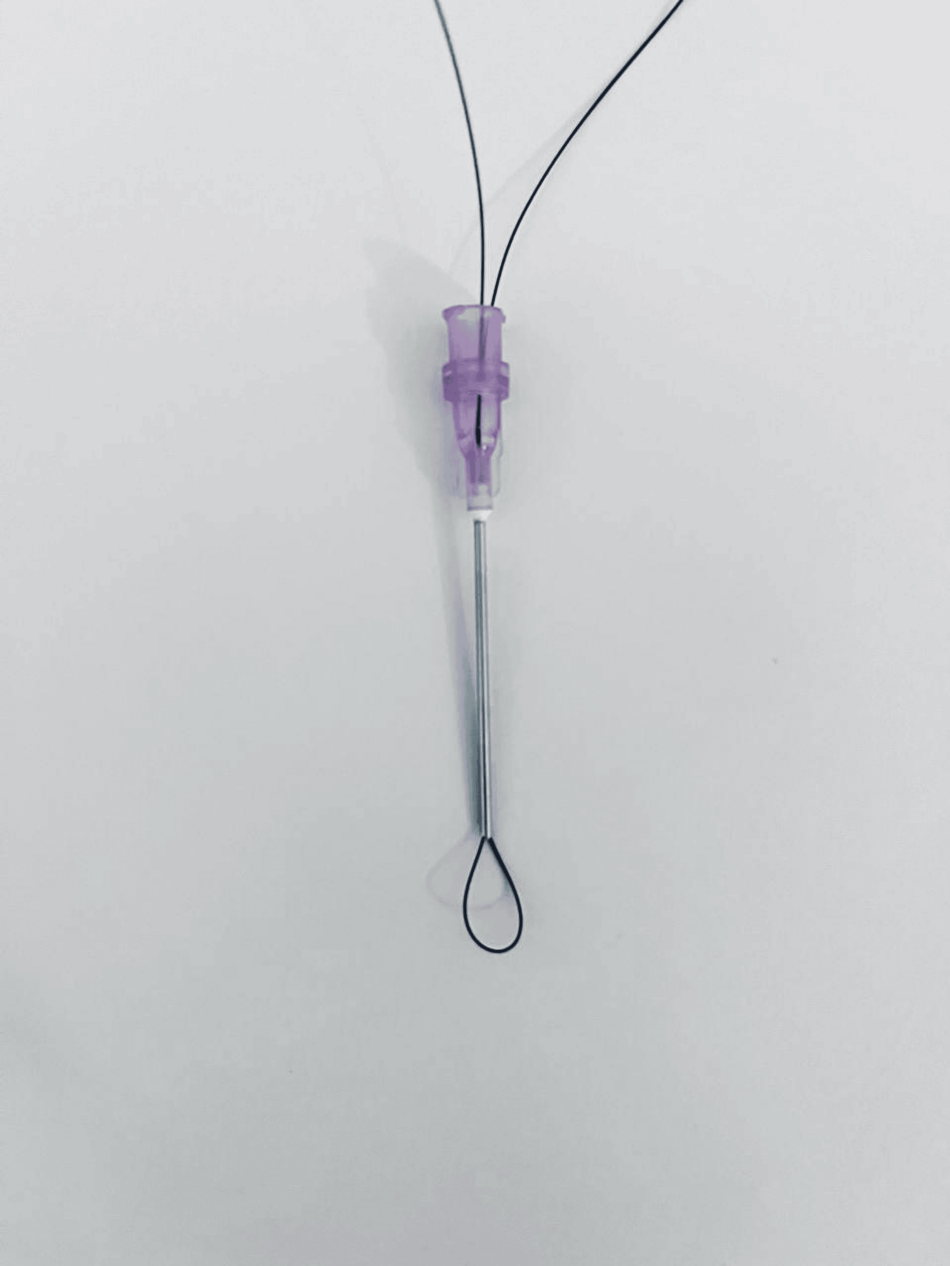
The final look of the suture passer after pulling both ends of the stitch.

This article describes the technique of using standard theatre materials to develop an innovative suture-passing technique for fixing patella fractures. The technique involves fashioning a suture passer by drawing the needle from a 2/0 nylon suture and pulling both ends through the insertion site using 18-gauge standard or spinal needles, depending on the length needed (Figures 1-3).

**Figure 4:**
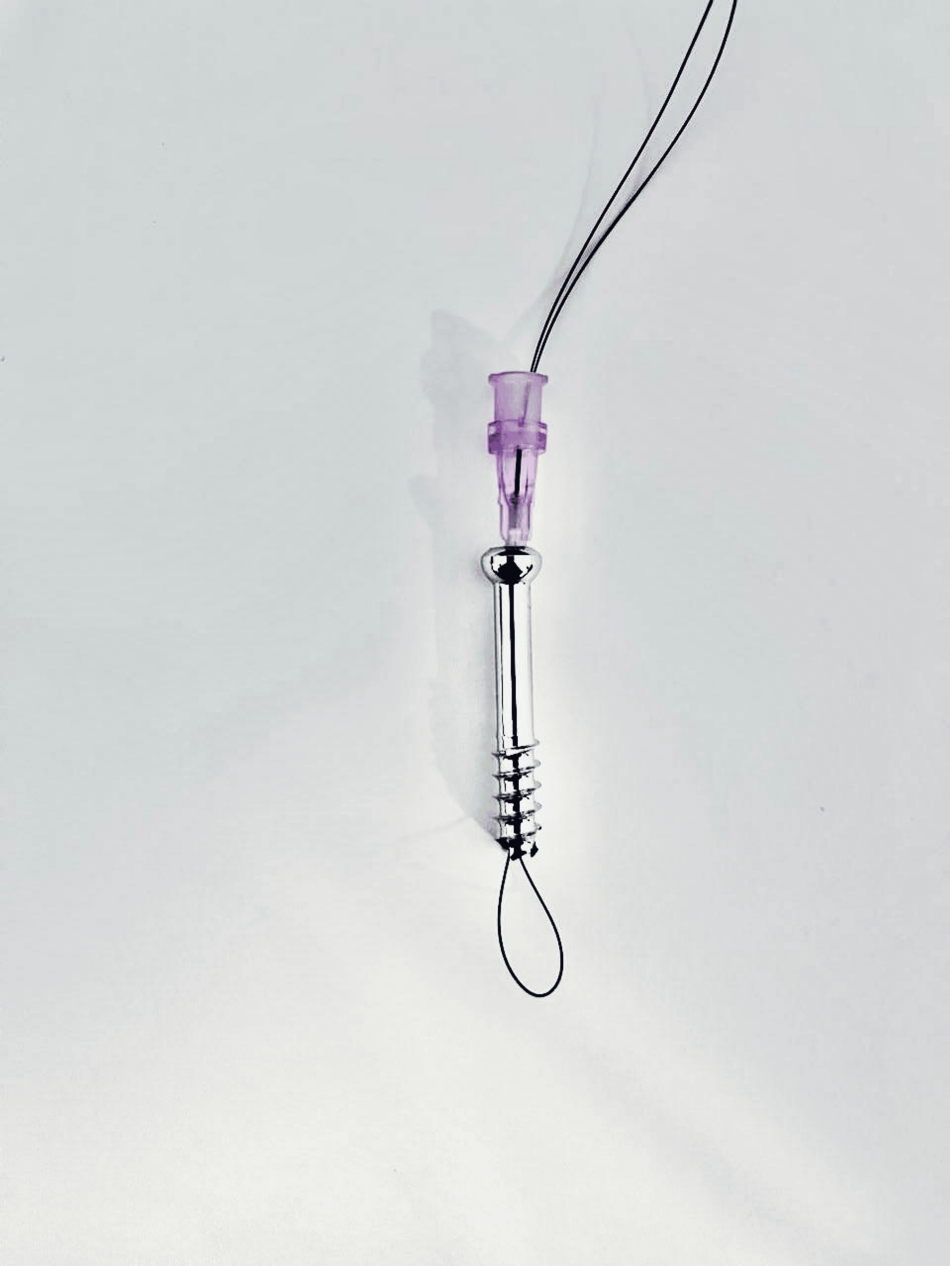
The suture passer through the cannulated screw.

**Figure 5:**
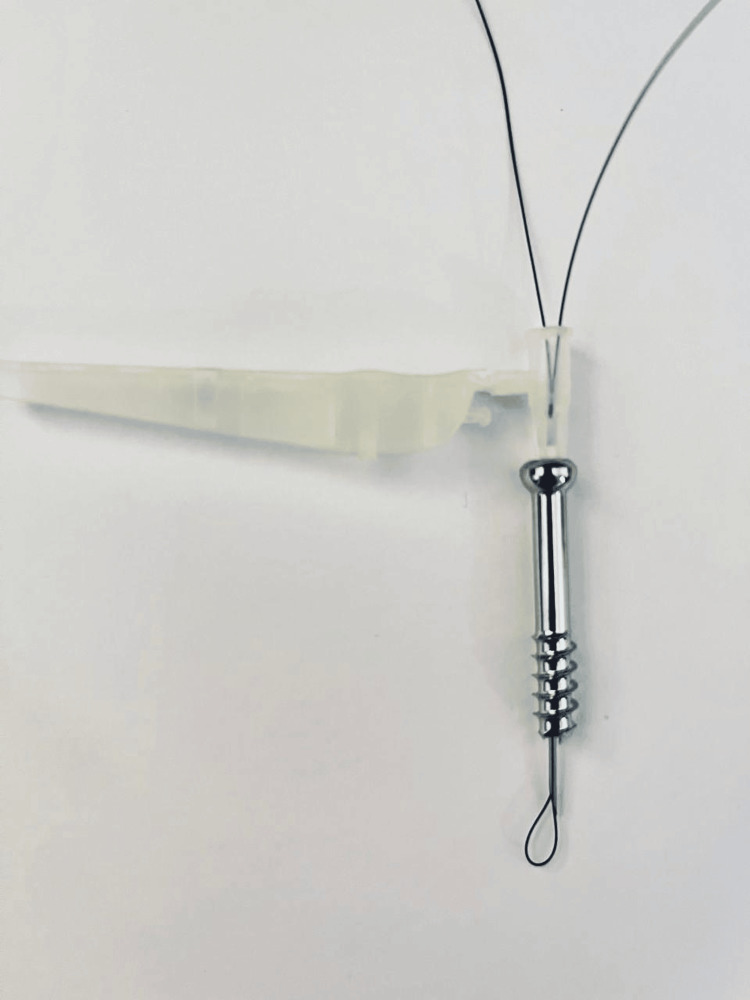
The same construct but using an 18-gauge white needle.

This construct functions as an effective suture passer capable of passing through 4.5-7.5 mm cannulated screws. It is a valuable technique, especially in patella fracture fixation with the FiberTape augmentation method. The modified suture passer is passed through the cannulated screw and osseous tunnel and then efficiently retrieves the FiberTape, pulling it along with it as a construct (Figures 4, 5).

**Figure 6:**
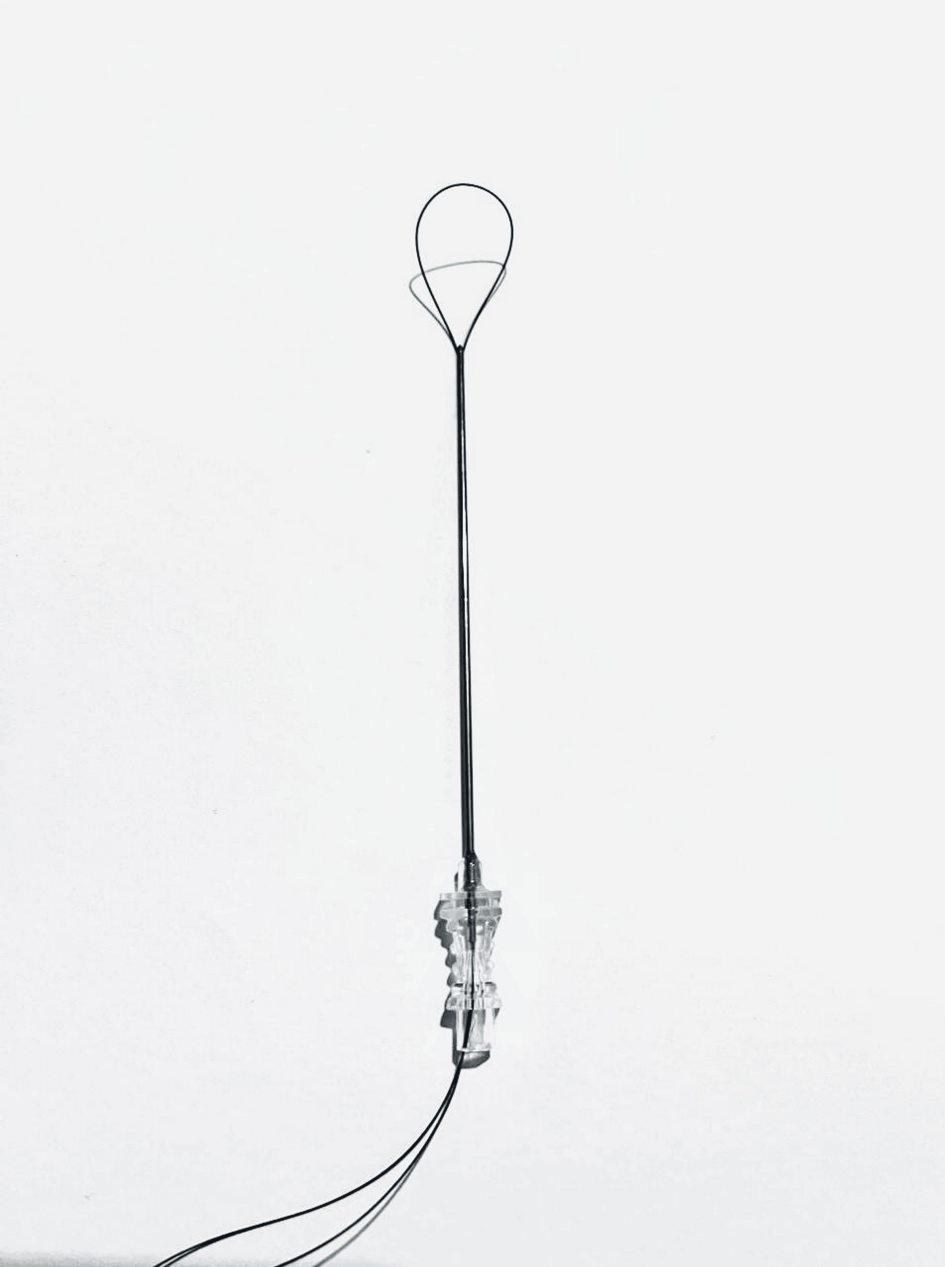
The suture passer employs an 18-gauge 90-mm spinal needle. This suture passer can be used in different clinical settings and offers additional length.

This technique may be applied across multiple lengths of needles and different sizes of cannulated screws, provided they are in the 4.5-7.5 mm range and adhere to the principles described within this text for using suture tape with standard cerclage fixation (Figure 6). This technique is a low-cost alternative to specific suture-passing instruments for an operating theatre and includes readily accessible materials.

## Discussion

More recently, the management of patellar fractures has evolved with new literature suggesting growing techniques in which rigid fixation is supplemented by suture augmentation [7].

Hoshino et al. showed that traditional tension band constructs had much higher complication rates of up to 37% for hardware irritation that required implant removal and 4.4% for post-op infection vs. lower implant-related complications in screw-only fixation. These results support the utility of augmented screw fixation over traditional methods of patella fixation (tension band construct) [8].

Although Arthrex has described the FiberTape augmented screw fixation technique, these techniques require the use of proprietary preparation and delivery systems that include specialized instrumentation, leading to added procedural costs. Conversely, we provide a cheap suture-passer technique suitable for all surgical settings [6].

The economic aspect of surgical instrumentation remains significant in orthopaedic procedures. Okike et al. found that while device costs constitute a substantial portion of surgical expenses, only 21% of surgeons could accurately estimate their costs [9]. Our technique addresses this challenge by utilizing readily available materials, which offer a cheap option of suture passer, especially in low-resource clinical settings, while maintaining modern fixation principles described in the literature [6,7,10].

## Conclusions

The Mo Passer technique is a useful innovation for patellar fracture fixation and serves as an economical, handy tip for specialized equipment. Using locally available materials and conforming to modern principles of fixation, this observance proves that surgery in austere environments can be effective with simple techniques.
